# 

**Published:** 1894-01

**Authors:** 


### INDEX

TO THE

### HOMŒOPATHIC PHYSICIAN.

### VOLUME XIV, 1894.

PAGE

**Aconite in Fever.** C. Carlton Smith, M. D. .... 321
**Aconite, The Use and Abuse of.** Walter M. James, M. D. .... 257
**Aconite** ....17, 20, 257
**A[illegible]ress to Delinquents in the December Number** .... 27
[illegible]ns ....17, 20
**Æsc[illegible]lus** ....17, 20
**Æt[illegible]sa.** .... 383
**Agaric** ....17, 20
**Ailanthus** ....18, 58
**Alexander III, Death of** .... 415
**Allan, Arthur G., M. D.** Chronic Dis[illegible]es and How to Manage them, 179
**A[illegible]an Institute of Homœopathy,** 228, 265, 268, 271, 272, 273, 414
**[illegible]erican Institute, Accommodations for the Next Meeting** .... 279
**American Institute of Homœopathy, The.** By Frank Kraft, M. D. Review of .... 220
**American Institute : Jubilee of** ....84, 216
**American Public Health Association.** Irving A. Watson, M. D. .... 288
**American Text-Book of Gynecology.** Review of .... 32
**Anatomist's Anomaly Blank, An.** Dr. A. Hewson. Review of .... 127
**Annual Reunion of the Alumni Association of the Hahnemann Medical College of Philadelphia.** Note .... 128
**Antidote to Cocaine.** J. G. Gundlach, M. D. .... 402
**Antidoting Crude Drugs with Potencies.** A. W. Holcombe, M. D. .... 217
**Apis-mel. (Bee Virus) for Acute Rheumatism** .... 63
**Apis-mel in Typhus.** Ad. Lippe, M. D. .... 40
**Apocynum** ....20, 58
**Appeal for Payment.** Walter M. James, M. D. .... 1
**Arans-excels.** .... 21
**Argent** .... 20
**Arsenic** ....57, 58
*Art Amateur, The.* Review of, 223, 255, 289, 335, 369
**Arum-tri** .... 21
**Aurum-mur** .... 21

PAGE

**Auto-intoxication, Lectures on.** Review of .... 333

**Bacon's, Sir Francis, Cipher Story,** 163, 186, 290
**Banerjee, Dr. D. N.** Tinaspora Cordifolia .... 367
**Baryta** .... 58
**Baylies, B. L. B., M. D.** Kind Words for the March Editorial .... 151
**Beach, Rev. Henry H., has Removed** ... 378
**Bedside Dietary, The.** By Gideon C. Segur. Review .... 192
**Belladonna.** Walter M. James, M. D. 295
**Bellad** .... 58, 295, 384
**Benzoic Acid** .... 385
**Berridge, E. W., M. D.** The Conditions of Nux Vomica in Whitlow, 28
**Bile Pigment** .... 421
**Bird's-Eye View of Bovista.** C. C. Smith, M. D. .... 381
**Bishop, H. D.** To the Medical Profession .... 375
**Bismuth in Cholera Infantum.** Clinical Observations, Ad. Lippe M. D .... 100
**Bœricke, Wm., M. D.** How Hahnemann Cured .... 137
**Boger, C. M., M. D.** Erotomania During Typhoid Fever .... 25
The Palliative Treatment of Pulmonary Tuberculosis .... 317
Three Cases of Intermittent Fever and How they were Treated .... 120
**Book Notices,** 29, 92, 122, 156, 186, 219, 254, 289, 333, 368, 417
**Borax** .... 21
**Bovista, A Bird's-Eye View of.** C. C. Smith, M. D .... 381
**Bread from Stones.** Review of .... 370
**Bromine** .... 21
**Bryonia** .... 387
**Bufo** .... 386
**Bunting, Thos. C., M. D.** Neuralgia in the Teeth .... 390
**Bursa-past** ....18, 21

**Cactus-grand** .... 17
**Campbell, J. B., M. D.** A "Refuse" Case .... 83
**Cancer of the Mammæ, cured by the Internal Remedy.** Discussion... 51

PAGE
Cannabis-ind ... 386
Carbolic Acid, The Antidote to. E. Carleton, M. D. ... 77
Carb-sulph ... 18, 21
Carleton, E., M. D. The Antidote to Carbolic Acid ... 77
Carleton, Edmund, M. D. A Case of Dermoid Cyst of the Parotid Gland, with Specimen ... 6
Staphylorraphy. A Case from Practice ... 4
Case, E. E., M. D. Clinical Experience with the CM Potency ... 383
Cases from Practice. Dr. Kunkel ... 140
Catlin, A. M., M. D. Diphtheria Treated with Antitoxin ... 410
Caust ... 18, 21
Chapman, Dr. Millie J. Removal of ... 128
Chelon-glab ... 18
Chicago Homœopathic Society ... 421
Childhood. Edited by Dr. Wm. Winterburn. Review of ... 94
Choate, Rufus, M. D. The Origin and Cure of Syphilis ... 199
Cholera Infantum, Bismuth in, Clinical Observations. Ad. Lippe, M. D. ... 100
Chorea ... 143
Chronic Diseases and How to Manage Them. Arthur G. Allan, M. D. ... 179
Cichor-intyb ... 18
Cimicifuga-rac ... 22, 58
Clarke, Dr. Note ... 375
Clinical Diagnosis. By Albert Abrams, M. D. Review of ... 334
Clinical Experiences, Some. J. Emmons, M. D. ... 323
Clinical Experience with the Single Dose of the CM Potency. E. E. Case, M. D. ... 383
Clinical Observations, Bismuth in Cholera Infantum. Ad. Lippe, M. D. ... 100
Cobalt ... 21
Cocaine, Antidote to. J. G. Gundlach, M. D. ... 402
Cochlearia ... 18, 22
Colic, Two Patients with. Charles G. Wilson ... 287
Committee on Legislation and the Promotion of Homœopathy ... 365
Complete Repertory of the Tissue Remedies of Schüssler. By S. F. Shannon, M. D. Review of ... 254
Concordance, Condensed. See Condensed Concordance.
Condensed Concordance of the Homœopathic Materia Medica, A. By J. G. Malcolm, M. D. Review of ... 223
Confederate Money. Note ... 336
Congenital Deformity, A Curious. Ella M. Tuttle, M. D. ... 150
Conservatism in Medicine. Walter M. James, M. D. ... 193
Cornus florid ... 16, 18
Correction ... 224
Correction in September Number. Wm. Keaney, M. D. ... 332
Cranch, Edward, M. D. The Law of Similars. Second Open Letter ... 196
Open Letter to a Physician who has Inquired about Homœopathy ... 131
A Grippe Characteristic ... 207
Physician's Library ... 406
Tuberculosis Alleged; Case Cured 286

PAGE
Cuprum-acet ... 17, 22, 58
Cystic Evacuations and Irrigation. By W. Storer How, D. D. S. Review of ... 157

Dake, J. P., M. D. In Memoriam ... 390
Dake, J. P., M. D.—Personal Recollections. B. W. James, M. D. ... 392
Daphne-ind ... 383
Davis, The F. A., Co. Note ... 293
Deformities and Diseases of Children .. 265
Deformity, Congenital, Curious. Ella M. Tuttle, M. D. ... 150
Delinquency of the Subscribers to THE HOMŒOPATHIC PHYSICIAN, The. Walter M. James, M. D. ... 259
Delinquents, Address to, in December Number ... 27
Denver Homœopathic Club ... 119
Dermoid Cyst of the Parotid Gland, with Specimen, A Case of. Edmund Carleton, M. D. ... 6
Dever, I., M. D. Metastasis of Gonorrhœa ... 80
Diagnosis, Clinical. Albert Abrams, M. D. ... 96, 334
Diagnosis, Differential, of Eyes ... 157
Dictionary of Medicine, illustrated. Review of ... 221, 256
Dictionary of Medicine. New Unabridged Pronouncing. Review of ... 94
Dictionary, Standard, of Funk & Wagnalls ... 30, 122, 191, 220, 255, 368, 420
Dietary, Bedside. By Gideon C. Segur. 192
Differential Diagnosis of Common Diseases of the Eyes. By W. F. Conners, M. D. Review of ... 157
Digit ... 59
Diphtheria Treated with Antitoxin. A. W. Catlin, M. D. ... 410
Diseases of the Hair and Scalp. By Geo. T. Jackson, M. D. Notice of 96
Diseases, The, of Inebriety from Alcohol, Opium, and Other Narcotic Drugs. Review of ... 372
Diseases of Woman. By Henry J. Garrigues, M. D. Review of ... 32
Dracontium ... 19
Drake, Olin M., M. D. Repertory of Foot Sweat ... 171
Dudley, Pemberton, M. D. The Jubilee of the American Institute ... 84

Editorials, 1, 33, 65, 97, 129, 161, 193, 225, 257, 295, 337, 379
Editorial in the February Number. C. Carleton Smith, M. D. ... 106
Electricity: Treatment by. Review of 289
Electrotherapeutists: Proceedings of ... 363
Emmons, Dr. J. Recovery of ... 256
Emmons, J., M. D. Some Clinical Experiences ... 323
Erecthites ... 22
Erotomania During Typhoid Fever. C. M. Boger, M. D. ... 25
Essentials of Homœopathic Materia Medica. By W. A. Dewey, M. D. Review of ... 125
Establishing a Method of Artificial Respiration in Asphyxia Neonatorum. By J. Harvie Dew, M. D. Review of ... 92
Eupat-perf ... 19, 22
Euthanasia. W. A. Yingling, M. D. ... 175
Examination Hom. Hospital ... 160

PAGE

Eye-openers, Some ... 332
Eye Symptoms, Selected. Mahlon Preston, M. D. ... 104

Fever: Aconite in. C. C. Smith, M. D. 321
Fincke, B., M. D. Commentaries on *The Organon* ... 35
Compulsory Vaccination ... 163
Fisher, Dr. C. E., President of the Institute ... 292
Foods, Patent ... 271
Foot Sweat, Repertory of. Olin M. Drake, M. D. ... 171
Forceps: How to Use the. Review of.. 96, 158
Formica ... 22
For Sale. No'e ... 292
Frederick Stearns & Co. Notice of Blotter ... 128
Funk & Wagnall's Standard Dictionary of the English Language, Review of ...30, 122, 191, 220, 255, 368, 420
Fun for Doctors ...294, 378, 422

Gelsem ... 22
Generalization: The Habit of. Walter M. James, M. D. ... 97
German-American Medical Journal, The. Review of ... 333
Gettysburg ... 19, 22
Glanderine ...19, 23
Glaucoma ... 61
Gleanings. F. H. Lutze, M. D. ..52, 86, 108, 152, 208
Glenmary Home. Note ... 292
Gonorrhœa, Metastasis of. I. Dever, M. D. ... 80
Graphites ...59, 385
Grippe, Characteristic, A. Edward Cranch, M. D. ... 207
Gundlach, J. G., M. D. Antidote to Cocaine ... 402
Gynæcology: American Text-book of.. 32

Hahnemann to Bœnninghausen, Two Cases Reported by ... 137
Hahnemann, Samuel. H. P. Holmes, M. D. ... 299
Hahnemann Cured: How. Wm. Bœricke, M. D. ... 137
Hahnemann Medical College of Phila. Annual Reunion ... 128
Hair and Scalp, Diseases of. Notice of 96, 417
Health Culture. Review of ... 419
Heart. Malcolm Macfarlan, M. D. ... 16
Heart, Morbid Conditions of ... 421
Hering, The Warning of Constantine. E. J. Lee, M. D. ... 69
Hesse, Dr. Sepia ... 276
Hint or two, A. C. Carleton Smith, M. D. ... 24
Holcombe, A. W., M. D Antidoting Crude Drugs with Potencies ... 217
Tuberculinum and Isopathy ... 82
Holmes, H. P., M. D. Samuel Hahnemann ... 299
Tuberculinum ... 8
Homœopath, How Shall We Dispose of the ... 160
Homœopathicians, The Society of ... 355
Homœopathic Club of Denver ... 119
Homœopathic Envoy. By E. P. Anshutz. Review of ... 334
Homœopathic Medical College of Missouri ...214, 388

PAGE

Homœopathic Medical Society of Ohio. Note ... 160
Homœopathic Medical Society of Chicago ... 421
Homœopathic Surgery. Wm. Keaney, M. D. ... 273
Homœopathy. Open Letter to a Physician Who Has Inquired About. Edward Cranch, M. D. ...131, 196
How Hahnemann Cured. William Bœricke, M. D. ... 137
How Shall We Dispose of the Homœopath. Note ... 160
How to Use the Forceps. By Charles H. Bushong, M. D. Review of... 158
By Prof. H. G. Landis, M. D. Notice of ... 96
Hydrogen Dioxide ... 376
Hygiene, Outlines of Practical. Review of ...29, 96
Text-Book of. Review of ... 374
Hypericum ...19, 23
Hypnotism in Surgery ... 268

Idealism in Medicine. Walter M. James, M. D. ... 161
Illustrated Dictionary of Medicine, Biology, and Allied Sciences. By George M. Gould, M. D. ...221, 256
Indiana, The Homœopathic Medical Society of ... 281
Individualization. Walter M. James, M. D. ... 129
Inebriety, The Disease of; from Alcohol, Opium, etc. Review of.. ... 372
In Memoriam. See "Memoriam, In."
Institute, The Jubilee of the American. Pemberton Dudley, M. D. 85
Instructions to Patients. By A. W. Holcombe, M. D. Review of ... 157
Intermittent Fever. Three Cases. C. M. Boger, M. D. ... 120
International Hahnemann Association ...192, 362
International Homœopathic Congress. 280
International Journal of Surgery, removal. Note ... 160
International Medical Annual for 1894. Review of ...31, 156
Intussusception Cured Without the Knife. Discussion on ... 48
Involuntary Proving of Fruit Wasp. C. F. Menninger, M. D. ... 403
Iodide-potassium ... 59
Iris ... 17
Ischias Sciatica ...141, 142
Isopathy. Walter M James, M. D. ... 2
Isopathy; Tuberculinum and ... 83

James, Bushrod W., M. D. Prof. J. P. Dake, M. D.—Personal Recollections ... 392
James, Walter M., M. D. Aconite, the Use and Abuse of ... 257
Appeal for Payment ... 1
Belladonna ... 295
Conservatism in Medicine ... 193
The Delinquency of the Subscribers to THE HOMŒOPATHIC PHYSICIAN ... 259
Facts in Homœopathic Prescribing 225
The Habit of Generalization ... 97
Idealism in Medicine ... 161
Individualization ... 129
Isopathy ... 2
The Potency Question ... 337

PAGE
James, Walter M., M. D. Reducing Temperatures... 379
The Responsibility of Selecting the Ideal Simillimum... 33
The Use and Abuse of Aconite... 257
Wells on Intermittent Fever... 259
A Word with Our Subscribers... 65

Kali-hyd... 17, 23, 59
Keaney, Wm., M. D. Correction in September Number... 332
Homœopathic Surgery... 273
Kinds Words for the March Editorial. B. L. B. Baylies, M. D... 151
Kitchen, James, M. D. In Memoriam, 262, 353
Kreosote... 59
Kunkel, Dr. Cases from Practice... 140

Lachesis... 59, 387
Law of Similars. Ed. Cranch, M. D.... 196
Lectures on Auto Intoxication in Disease or Self-poisoning of the Individual. By Ch. Bouchard... 333
Ledyard. W. E., M. D. The Organon and Materia Medica Club of the Bay Cities of California... 324
Lee, E. J., M. D. The Warning of Constantine Hering... 69
Legislation, Committee on... 365
Letters, Open, to a Physician. Edward Cranch, M. D... 131, 196
Liberality in Medicine. C. H. Oakes, M. D... 260
Lippe, Ad., M. D. Apis-mel in Typhus. 40
Clinical Observations; Bismuth in Cholera Infantum... 100
Lithium-carb... 23, 59
Lobelia... 59
Longevity. By Archer E. Atkinson, M. D. Review of... 127
Lutze, F. H., M. D. Gleanings...52, 86, 108, 152, 208
Lyc... 59

Mcfarlan, Malcolm, M. D. Provings and Clinical Observations, with High Potencies... 16, 56
Manual of Clinical Diagnosis. By Albert Abrams, M. D. Notice of, 96, 334
Materia Medica, Essentials of. Notice of... 125
McKay, Dr. Jean Ida. Note... 292
McNeil, A. Relation of Pain to Swallowing... 62
Medical Annual, International, for 1894... 31, 156
Medical Publishers, To. Note... 256
Medicine. J. M. Selfridge, M. D... 339
Medicine, Legal, System of... 95, 219
Medicine, Liberality in. C. H. Oakes, M. D... 260
Medicine, Theory and Practice of, Text-Book of. Review of... 95, 418
Meeting of Medical Publishers. Note. 336
Memoriam, In. J. P. Dake, M. D... 390
James Kitchen, M. D... 262, 353
Dr. John C. Robert... 28
Julius G. Schmitt, M. D... 146
Samuel Swan, M. D... 26
Dr. Lucien B. Wells... 149, 206
Menninger. C. F., M. D. Involuntary Proving of Fruit Wasp... 403
Mercury, The Use of... 273
Merc-prot-jod... 23, 60

PAGE
Merc-sol... 60
Merc-viv... 19, 23
Metropolitan Post-Graduate School... 420
Missouri Institute of Homœopathy. Note... 96
Murdered by Allopathy... 252
Musical Contest. Note... 32
Myrtus... 19

Natrum-mur... 60
Nervous Exhaustion. Practical Treatise on. Review of... 371
Neuralgia in the Teeth. Thos. C. Bunting, M. D... 390
New Unabridged Pronouncing Dictionary of Medicine. By John M. Keating, M. D. Review of... 94
New York Homœopathic Union... 69, 143
Nitric-acid... 24
Northern Indiana and Southern Michigan Society, The... 128, 360
Notes and Notices, 32, 64, 96, 128, 160, 192, 195, 224, 256, 292, 335, 375, 420
Numerical Strength of the Different Schools of Medicine. Note... 293
Nux-vomica, The Conditions of in Whitlow. E. W. Berridge, M. D. 28
Nux Case. A Model Cure. G. J. Waggoner, M. D... 122

Oakes, Dr. C. H., has Removed... 378
Liberality in Medicine... 260
Oh! Ye of Little Faith. C. Carleton Smith, M. D... 351
*Organon*, Commentaries on the. B. Fincke, M. D... 35
Organon and Materia Medica Club of the Bay Cities of California. W. E. Ledyard, M. D... 293, 324, 397
Origin and Cure of Syphilis. Rufus Choate, M. D... 199
Outlines of Practical Hygiene, Adapted to American Conditions. By C. Gilman Currier, M. D. Review of... 29

Palliative Treatment of Pulmonary Tuberculosis. C. M. Boger, M. D. 317
Patent Foods... 271
Peck, Geo. B., M. D. American Institute of Homœopathy... 414
Penfield, Sophia, M. D. A Woman's International Provers' Association... 331
People's Health Journal, The. Review of... 335
Petroleum... 24, 60
Pharmaceutical Era for May. Notice of 223
Philanthropic Index and Review. Notice of... 419
Phillips, Dr. Walter H., has Removed, 336
Photographic Panorama of the World's Fair. Notice of... 126
Physician's Bedside Record with Dietary. By Gideon C. Segur, M. D. Review of... 192
Physician's Library. E. Cranch, M. D. 406
Physician's Visiting List, The. Lindsay & Blakiston. Review of... 30, 419
Physician's Wife, The. By Ellen M. Firebaugh. Review of... 126
Phytolacca... 19, 24, 60
Plantago-min... 19, 24
Polygonum... 24
Post-Graduate School, Metropolitan... 420
Potencies, Antidoting Crude Drugs with. A. W. Holcombe, M. D... 217

PAGE

Potencies, Proving and Clinical Observations with High. M. Macfarlan, M. D. ... 16, 56
Potency, CM; Clinical Experience with. E. E. Case, M. D. ... 383
Potency Question, The. Walter M. James, M. D. ... 337
Powel, Dr. Milton. Removal ... 375
Powel, Dr. Wm. R., has Removed ... 378
Practical Hygiene. C. G. Currier, M. D. Notice of ... 96
Practical Treatise on Nervous Exhaustion. By George M. Beard, M. D. Review ... 371
Practical Uranalysis and Urinary Diagnosis. By Charles W. Purdy, M. D. Review of ... 254
Prescribing, Facts in Homœopathic. Walter M. James, M. D ... 225
Preston, F., M. D. Waiting Upon the Remedy ... 409
Scarlatina and Variola ... 413
Preston, Mahlon, M. D. Selected Eye Symptoms ... 104
Proceedings of the American Institute. Selected Extracts ... 228, 265, 268, 271, 272, 273
Proceedings of the Society of Electro-therapeutists ... 363
Professional Repartee. Note ... 32
Provers' Association, Woman's International ... 331
Proving of Fruit Wasp. Involuntary .. 403
Provings and Clinical Observations with High Potencies. Malcolm Macfarlan, M. D. ... 16, 56
Psorin ... 56

Quackenbush, A., M. D. Renal Colic, 102

Ranunculus-acris ... 20, 56
Reducing Temperature in Fevers. Editorial ... 379
"Refuse" Case, A. J. B. Campbell, M. D. ... 83
Reininger, Dr. E. E., has Removed ... 378
Relation of Climate and Altitude to Insanity ... 272
Removal ... 163
Renal Colic. A. Quackenbush, M. D.. 102
Respiration Artificial in Asphyxia Neonatorum. Notice of. ... 92
Responsibility of Selecting the Ideal Simillimum, The. Walter M. James, M. D. ... 33
Rheumatism. Bee Virus for Acute ... 63
Rhus Poisoning, How to Cure ... 29
Rhus-tox ... 56, 60
Ringworm ... 15
Roberts, Dr. Thomas G., has Removed.. 378
Robert, Dr. John C. In Memoriam ... 28
Rosedale. Note ... 335
Rumex ... 20

Sabina ... 385
Saliva. Cold Remedies Having. W. A. Yingling, M. D. ... 64
Sambucus ... 57
Sanders, Mrs. Albina G. Smith, Death of. Notice ... 293
Sap soda ... 56
Sassafras ... 57
Satterlee, Dr., M. D., has Removed ... 378
Scarlatina and Variola. F. Preston. 413
Schmitt, Julius G., M. D. In Memoriam 146
Scientific American. Review of ... 419
Scientific American Architects' and Builders' Edition. Review of... 126
Scrofula, Discussion on ... 43
Scudder, J. M., M. D., Death of. Note.. 128
Secale ... 57, 58, 61
Selfridge, J. M., M. D. & C. M., M. D., co-partners. Note ... 96
Selfridge, J. M., M. D., Medicine ... 339
Sepia. Dr. Hesse ... 276
Sepia ... 276, 278
Significant Notes ... 62
Silicea, Surgical Value of ... 47
Silicea ... 20, 47, 61
Similars, The Law of Edward Crauch, M. D. ... 196
"Simillimum in Surgery." Discussion 51
Sir Francis Bacon's Cipher Story. By Orville W. Owen, M. D. Review of ... 163, 186, 290
Skinner, Thomas, M. D. In Memoriam, Samuel Swan, M. D. ... 26
Skinner, Dr. Thomas, has Removed. Note ... 192
Smith, C. Carleton, M. D. Aconite in Fever ... 321
The Editorial in the February No., 106
A Hint or Two ... 24
"Oh! Ye of Little Faith" ... 351
Bird's-Eye View of Bovista ... 381
Southern Pines, Moore Co., N. C. Note 32
Spigelia ... 17
Standard Dictionary of English Language, A. By Funk & Wagnalls. Review of ... 30, 122, 191, 220, 255, 368, 420
Staphylorraphy: A Case from Practice. Edmund Carleton, M. D. ... 4
Stearns, F. & Co., Blotter ... 128
Stearns' Calendar for 1894. Note ... 64
St Louis *Clinique*, The. Note ... 256
Strike at Shane's, The. Review of ... 93
Strontia-carb. ... 17, 56
Subscribers, A Word with our. Walter M. James, M. D. ... 65
Sulphur ... 56, 61
Surgery, Discussion upon ... 47
Hypnotism in ... 268
Simillimum in ... 51
Surgery, International Journal of ... 160
Surgical Cases Cured by the Indicated Remedy. Discussion on ... 49
Surgical Value of Silicea ... 47
Swallowing. Relations of Pain to. A. McNeil, M. D. ... 62
Syllabus of Lectures on Human Embryology. By Walter Porter Manton, M. D. Review of ... 373
Synopsis of Practice ... 418
Syphilis. The Origin and Cure of. Rufus Choate. M. D ... 199
System of Legal Medicine, A. By Allan McLane Hamilton, M. D. Review of ... 95, 219

Taraxacum ... 20
Tart-emet ... 61
Teeth, Neuralgia in. Thos. C Bunting, M. D. ... 390
Temperature in Fever, Reducing ... 379
Terebinth ... 16, 58
Text-book of Hygiene. By George H. Rohé, M D. Review of ... 374
Text-book of the Theory and Practice of Medicine by American Teachers. Edit. by William Pepper, M. D. Review of ... 95

PAGE
Thompson, J. W., M. D. In Memoriam. Dr. John C. Robert ... 28
Tinaspora Cordifolia. Dr. D. N. Banerjee ... 367
Tissue Remedies of Schüssler. Complete Repertory of ... 254
To the Medical Profession. H. D. Bishop ... 375
Treatment by Electricity. By Nondo Lal Ghose, L. M. S. Review of... 289
Trillium-pend ... 16
Triticum ... 16, 61
Trombidium ... 16, 57
Tubercullnum. Horace P. Holmes, M. D ... 8
Tuberculinum and Isopathy. A. W. Holcombe, M. D ... 83
Tuberculosus Alleged; Case Cured. Edward Cranch, M. D. ... 286
Tuberculosis, The Palliative Treatment of Pulmonary. C. M. Boger, M. D ... 317
Tuttle, Ella M., M. D. A Curious Congenital Deformity ... 150
Two Cases Reported by Hahnemann to Bœnninghausen ... 137
Typhoid Fever, Erotomania during... 25
Typhus, Apis mel. in ... 40

Uranalysis, Practical. Review of ... 254

Vaccination, Compulsory. B. Fincke, M. D ... 163
Died of ... 274
No Safeguard ... 283
A Result of ... 85
Vandergrift's Hand-book of the United States Tariff. Review of ... 224, 291
Variola and Scarlatina ... 413

PAGE
Variolin ... 61
Venereal and Urinary Diseases. By Temple S. Hoyne, M. D. Review of ... 156
Verbascum ... 57
Vermiform Appendix ... 375
Vick's Floral Guide. Review of ... 94
Visiting List ... 30, 419

Waggoner, G. J., M. D. A Nux Case; "Model Cure." ... 122
Waiting upon the Remedy. F. Preston, M. D ... 409
Warning of Constantine Hering, The. E. J. Lee, M. D ... 69
Washington State Homœopathic Medical Society ... 184
Wasp, Fruit; Involuntary Proving of.. 403
Watson, Irving A., M. D. American Public Health Association ... 288
Wells, Dr. L. B. In Memoriam, 149, 206, 421
Wells on Intermittent Fever. Walter M. James, M. D ... 259
Wilcox, Emma D., M. D. The New York Homœopathic Union ... 143
Wilson, Chas. G., M. D. Two Patients with Colic ... 287
Woman, Diseases of. Review of ... 32
Woman's International Provers' Association, A. Sophia Penfield ... 331
World's Fair, Photographic Panorama of ... 126

Yale Surgical Chair, The. Note ... 224
Yingling, W. A., M. D. Euthanasia... 175
Remedies Having Cold Saliva ... 64

Zinc ... 16

VOL. XIV. JANUARY, 1894. No. 1.

### THE HOMŒOPATHIC PHYSICIAN,

A Monthly Journal of Homœopathic Materia Medica and Clinical Medicine.

*"He is a freeman whom truth makes free, and all are slaves beside."*

*"Seek the Truth: come whence it may, cost what it will."*

EDITED AND PUBLISHED BY

WALTER M. JAMES, M. D.

NOTICE.

***All articles for publication, books for review, business communications, etc., must be sent to Editor, 1125 Spruce Street, Philadelphia.***

***Subscribers will please notice that this Journal will be sent until arrears are paid and it is ordered discontinued.***

PHILADELPHIA:

